# Selenoproteins: Minute yet vital players governing cellular fate

**DOI:** 10.1016/j.gendis.2025.101938

**Published:** 2025-11-14

**Authors:** Chaoyi Xia, Yifan Wu, Haoming Zhang, Lang Qin, Yiren Hu, Caiyun Fu

**Affiliations:** a Zhejiang Provincial Engineering Research Center of New Technologies and Applications for Targeted Therapy of Major Diseases, College of Life Sciences and Medicine, Zhejiang Sci-Tech University, Hangzhou, Zhejiang 310018, China; b The Third Clinical College of Wenzhou Medical University (Wenzhou People's Hospital), Wenzhou, Zhejiang 325000, China

**Keywords:** Cancer, Ferroptosis, SECIS, Selenocysteine, Selenomethionine, Selenoproteins

## Abstract

Selenoproteins represent a distinct class of proteins that incorporate selenocysteine (Sec), whose biosynthesis and translational integration are dependent on selenium availability and the presence of a selenocysteine insertion sequence (SECIS). These proteins are indispensable for redox regulation, antioxidant defense, and thyroid hormone metabolism, among other vital biological processes. Remarkably, selenoproteins act as critical regulators of cellular fate decisions, a function that hinges on Sec-a residue whose biosynthesis and translational incorporation into protein involve machinery far more intricate than that of canonical amino acids. This evolutionary adaptation, whether arising from stochastic mutational events or as an obligatory trade-off for functional precision, underscores the sophisticated molecular regulatory strategies in living organisms. In this review, we comprehensively outline the uptake and metabolic pathways of selenoamino acids in eukaryotes, with particular emphasis on the biosynthetic mechanism of Sec and its unique translational incorporation into selenoproteins. We systematically elucidate the multi-layered regulatory networks that govern these biological processes within cells. Furthermore, we present a taxonomic classification and functional synthesis of eukaryotic selenoproteins, accompanied by an in-depth analysis of their molecular roles in various pathological states. Special emphasis is placed on the glutathione peroxidase (GPX) family, especially GPX4, in ferroptosis regulation and its sophisticated control mechanisms. Additionally, this review summarizes key challenges in current selenoproteins research and explores potential therapeutic strategies for cancer treatment by targeting selenoproteins.

## Introduction

The discovery of the DNA double helix,^1^ the formulation of the central dogma,^2^ and the deciphering of the genetic code^3^, ^4^, ^5^, ^6^ stand as the three landmark achievements of 20th-century molecular biology. Among these, the elucidation of the genetic code during the 1960s is widely regarded as the crowning accomplishment, representing a pinnacle of scientific rigor in the era. The deciphering process can be unfolded in two distinct phases: the mathematical reasoning stage in the 1950s and the experimental validation stage from 1961 to 1965. The discovery of transfer RNA (tRNA), a critical adaptor molecule linking mRNA to protein synthesis, provided substantial support for the central dogma and significantly advanced the deciphering of the genetic code.^7^, ^8^, ^9^, ^10^ Concurrently, tRNA research laid the foundation for understanding selenoproteins' biology.

**Figure 1:**
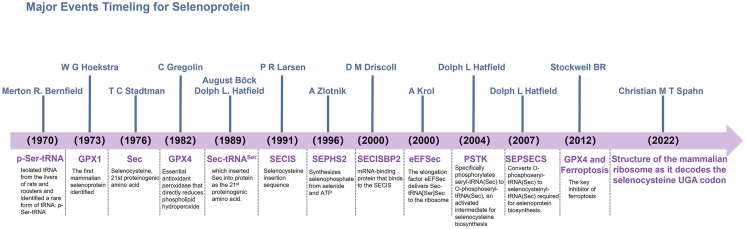
The history and development of selenoprotein (the major events in the field of selenoprotein from 1970 to 2022).

In 1970, Merton R. Bernfield isolated tRNA from the livers of rats and roosters and identified a rare form of tRNA: p-Ser-Trna.^11^ Simultaneously, Franklin H. Portugal identified a Ser-tRNA in rabbit and chicken tissues capable of recognizing the stop codon UGA.^12^ For over a decade, the mechanistic basis of UGA recognition by Ser-tRNA remained enigmatic.^13^ This problem was resolved in 1982 when Bernard Dudock demonstrated that the Ser-tRNA recognizing UGA and p-Ser-tRNA were the same molecule.^14^ Parallel studies revealed that the activity of certain enzymes depended on selenium.^15^, ^16^, ^17^, ^18^ Using selenium isotopes in labeling experiments, they detected selenium incorporation into both proteins and tRNA.^19^ However, the critical link between p-Ser-tRNA (UGA recognition) and the covalent incorporation of selenium into both polypeptides and tRNA remained elusive. A pivotal breakthrough occurred in 1988 when August Böck identified a gene encoding a unique tRNA capable of recognizing the stop codon UGA and serine while incorporating selenocysteine into proteins.^20^ Subsequently, Böck and Hatfield independently confirmed that p-Ser-tRNA, which recognizes UGA, was in fact sec-tRNA^ser^, and mediates the insertion of selenocysteine (Sec) into proteins.^21^^,^^22^ Thus, Sec was identified as the 21st proteinogenic amino acid.^23^ Since then, research in selenoproteins biology has coalesced around four central themes: i) intracellular selenium metabolism and regulation, ii) Sec biosynthesis and its regulatory networks, iii) mechanisms of Sec incorporation into proteins, and iv) functional characterization of selenoproteins (Fig. 1).

In this review, we systematically dissect the metabolic pathways of selenium and selenium-containing amino acids, selenomethionine (SeMet) and Sec, as well as their regulatory mechanisms. Next, we delineate the biosynthesis of Sec from serine and the intricate regulatory layers governing this process. Special emphasis is placed on the unique translational machinery enabling Sec incorporation, with a focus on the molecular discrimination between Sec incorporation and UGA termination codons. Furthermore, we provide a comprehensive overview of the identification and classification of human selenoproteins and their physiological functions, as well as emerging roles in disease pathogenesis. Particular attention is devoted to glutathione peroxidase 4 (GPX4), a critical selenoprotein involved in ferroptosis, highlighting its regulatory mechanisms and therapeutic implications. Finally, we trace the evolutionary origins of selenoprotein machinery and explore translational opportunities in cancer therapeutics.

## Acquisition and metabolism of selenium

**Figure 2:**
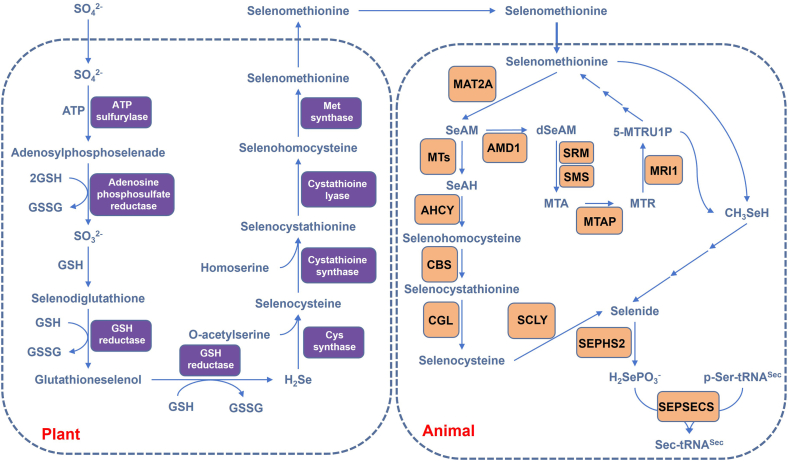
Metabolic processes of selenium-containing amino acids in plants and animals. In the figure, we described the *de novo* synthesis process of selenomethionine in plants and its catabolic metabolism as the main source of selenium in animals. MAT2A, S-adenosylmethionine synthase isoform type-2; MTs, methyltransferase; AHCY, adenosylhomocysteinase; CBS, cystathionine beta-synthase; CGL, cystathionine gamma-lyase; AMD1, S-adenosylmethionine decarboxylase 1; SRM, spermidine synthase; SMS, spermine synthase; MTAP, S-methyl-5′-thioadenosine phosphorylase; MRI1, methylthioribose-1-phosphate isomerase; SCLY, selenocysteine lyase; SEPHS2, selenide, water dikinase 2; SEPSECS, O-phosphoseryl-tRNA(Sec) selenium transferase.

Selenium exists primarily in the chemical forms of SeMet,^24^, ^25^, ^26^, ^27^ Sec,^28^ selenate, and selenite, with SeMet being the predominant form. Over 90% of selenium in plants is present as SeMet, and this compound also serves as the primary source of selenium for mammals.^29^^,^^30^ Interestingly, plants are among the few organisms lacking canonical selenoproteins; selenoproteins in plants are produced by incorporating Sec and SeMet into proteins nonspecifically through the metabolic pathway of sulfur analogues, thereby replacing methionine and cysteine.^31^^,^^32^ The SeMet stored in plants is not a naturally occurring amino acid in nature, but is synthesized by plants through the *de novo* methionine biosynthesis pathway.^33^^,^^34^ Plants initially absorb SeO_4_^2−^ from soil, which is then catalyzed by ATP sulfurylase to form adenosylphosphoselenate in the presence of ATP.^35^^,^^36^ Subsequently, adenosine phosphosulfate reductase, utilizing glutathione (GSH) as a reducing agent, converts adenosylphosphoselenate into SeO_3_^2−^.^37^, ^38^, ^39^ This selenite further reacts with GSH to produce selenodiglutathione, which is then repeatedly reduced by glutathione reductase to generate hydrogen selenide (H_2_Se).^33^^,^^40^ Notably, H_2_Se is a critical intermediate in the conversion of inorganic selenium into selenoamino acids in plants, a process analogous to sulfur assimilation. Following this, plants utilize the *de novo* methionine biosynthesis pathway to synthesize SeMet. First, cysteine synthase catalyzes the reaction between O-acetylserine and H_2_Se to produce Sec.^41^ Next, cystathionine γ-synthase facilitates the condensation of Sec with homoserine, yielding selenocystathionine.^33^^,^^42^ This intermediate is then cleaved by cystathionine β-lyase to form selenohomocysteine.^43^ Finally, selenohomocysteine is converted into SeMet under the catalysis of methionine synthase^44^ (Fig. 2).

SeMet, abundant in animal and plant proteins, serves as the main dietary selenium source for humans.^45^ Free SeMet can be derived either from pre-existing free SeMet in food or through the proteolytic degradation of SeMet-containing dietary proteins. In mammals, SeMet is metabolized via the transsulfuration pathway to produce Sec.^46^, ^47^, ^48^ This process involves several enzymatic steps: First, SeMet is converted by methionine adenosyltransferase into Se-adenosylmethionine (SeAM).^47^^,^^49^ Subsequently, methyltransferases catalyze the demethylation of SeAM, yielding Se-adenosylhomocysteine (SeAH). SeAH is then hydrolyzed by Se-adenosylhomocysteine hydrolase to form selenohomocysteine (SeHcy). In the next step, cystathionine-β-synthase (CBS)^50^^,^^51^ mediates the conversion of SeHcy into selenocystathionine, which is further cleaved by cystathionine γ-lyase (CGL)^52^ to generate Sec.

Notably, free Sec cannot be directly incorporated into selenoproteins. Instead, it is degraded by selenocysteine lyase (SCLY)^53^ into selenide, which is subsequently utilized for the *de novo* synthesis of Sec on Ser-tRNA^Sec^.^54^, ^55^, ^56^ Alternatively, CGL can also catalyze the decomposition of SeMet into methylselenol,^57^^,^^58^ which may be remethylation to form dimethylselenide, contributing to selenium pools for Sec biosynthesis.^45^ Given their structural similarity, SeMet may also utilize the methionine salvage pathway for its catabolism, ultimately supplying selenium in the form of selenide for cellular utilization^47^^,^^59^ (Fig. 2).

## Synthesis process of selenocysteine

**Figure 3:**
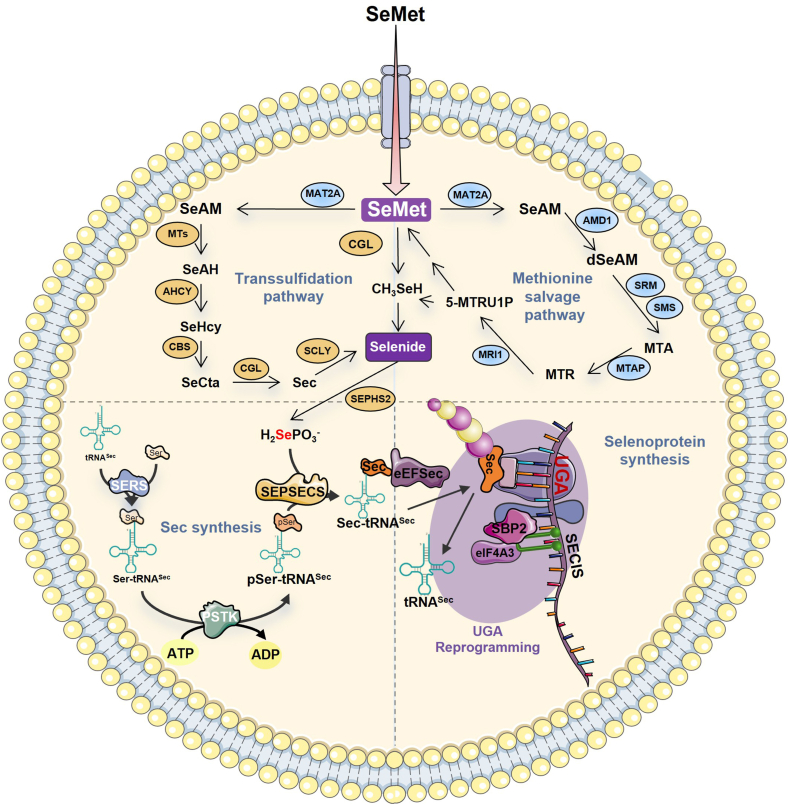
The synthesis process of selenoproteins. In the figure, we described the *de novo* synthesis process of selenocysteine (Sec) and its insertion into selenoproteins, as well as its regulatory process. SeMet, selenomethionine; MAT2A, S-adenosylmethionine synthase isoform type-2; MTs, methyltransferase; AHCY, adenosylhomocysteinase; CBS, cystathionine beta-synthase; CGL, cystathionine gamma-lyase; AMD1, S-adenosylmethionine decarboxylase 1; SRM, spermidine synthase; SMS, spermine synthase; MTAP, S-methyl-5′-thioadenosine phosphorylase; MRI1, methylthioribose-1-phosphate isomerase; SCLY, selenocysteine lyase; SEPHS2, selenide, water dikinase 2; SEPSECS, O-phosphoseryl-tRNA(Sec) selenium transferase; PSTK, L-seryl-tRNA(Sec) kinase; SERS, serine-tRNA ligase; eEFSec, selenocysteine-specific elongation factor; eIF4A3, eukaryotic initiation factor 4A-III; SBP2, selenocysteine insertion sequence-binding protein 2; SECIS, selenocysteine insertion sequence.

Although both are selenium-containing amino acids, Sec differs from SeMet in that it exists in two distinct forms: free Sec and Sec covalently linked to tRNA (Sec-tRNA^Sec^).^60^, ^61^, ^62^ Notably, free Sec does not participate in selenoprotein synthesis. As the 21st proteinogenic amino acid, Sec biosynthesis in eukaryotes exhibits distinct mechanistic features compared with canonical amino acids.^20^^,^^63^^,^^64^ The synthesis of Sec in eukaryotes involves four key steps: i) Aminoacylation: Seryl-tRNA synthetase (SerRS) catalyzes the attachment of serine to tRNA^Sec^, forming Ser-tRNA^Sec^.^65^, ^66^, ^67^ This step relies on the unique structural recognition of tRNASec by SerRS, distinguishing it from canonical serine tRNAs. ii) Phosphorylation: Phosphoseryl-tRNA^Sec^ kinase (PSTK) phosphorylates Ser-tRNA^Sec^ to produce pSer-tRNA^Sec^.^68^^,^^69^ This modification primes the tRNA for subsequent selenium incorporation. iii) Selenium activation: Selenophosphate synthetase (SPS) converts selenide into the active selenium donor, hydrogen selenophosphate (H_2_SePO_3_^−^).^70^^,^^71^ This reaction represents a rate-limiting step in Sec biosynthesis, tightly regulated by cellular selenium availability. iv) Selenocysteine formation: Selenocysteine synthase (SecS) catalyzes the reaction between H_2_SePO_3_^−^ and pSer-tRNA^Sec^ to generate Sec-tRNA^Sec^^63^^,^^72^^,^^73^ (Fig. 3). Interestingly, sulfide can substitute for selenide in this pathway. Selenophosphate synthetase can also catalyze sulfide to produce hydrogen thiophosphate (H_2_SPO_3_^-^), which, in turn, reacts with pSer-tRNA^Sec^ to form Cys-tRN^Sec^.^74^^,^^75^ This phenomenon arises from the structural similarity between cysteine and selenocysteine.

Sec is a selenium-containing analogue of cysteine (Cys) and occurs in selenoproteins across all three domains of life-bacteria, archaea, and eukaryotes. Selenoproteins exploit the shared chemical properties of Sec and Cys during their biosynthesis, with Sec often residing at catalytic sites. Although isosteric, Sec and Cys differ in key elemental characteristics, conferring upon Sec distinct biochemical behaviors under physiological conditions.

Compared with sulfur (S), selenium (Se) exhibits higher polarizability, rendering it a superior nucleophile and electrophile in substitution reactions. The selenol group of Sec has a lower pKa than the thiol group of Cys (5.5 *vs*. 8.7), which implies that Sec remains largely deprotonated at physiological pH, thereby enhancing its nucleophilic capacity. Furthermore, Sec displays a lower reduction potential relative to Cys, making the selenol group more susceptible to oxidation upon aerial exposure and more resistant to reduction.

Capitalizing on the chemical resemblance between Sec and Cys, Sec has been widely employed in protein chemical synthesis. The inefficient recombinant production of selenoproteins, due in part to differences in incorporation mechanisms between bacterial and mammalian systems, has made chemical (or semisynthetic) approaches a versatile alternative for obtaining homogeneous selenoproteins.

## The unique translational mechanism of selenoproteins

A precise definition of selenoproteins is that their biosynthesis requires the incorporation of Sec, which overrides the canonical termination function of the UGA stop codon. As the 21st proteinogenic amino acid,^23^ Sec is incorporated into proteins through a distinct translational mechanism compared with the other 20 amino acids.^76^, ^77^, ^78^ In selenoprotein mRNAs, the universally recognized UGA termination codon is reprogrammed for Sec insertion, a process orchestrated by an elaborate regulatory network in eukaryotes.^61^^,^^79^^,^^80^ To ensure that UGA encodes Sec exclusively in selenoproteins and not in other proteins, eukaryotes have evolved specialized machinery.^81^, ^82^, ^83^, ^84^ Central to this mechanism is the selenocysteine insertion sequence (SECIS), a conserved RNA stem-loop structure embedded within the 3′-untranslated region (3′-UTR) of selenoprotein mRNAs.^61^^,^^85^^,^^86^ This cis-acting element serves as a molecular beacon, ensuring that only UGA codons within selenoprotein-coding transcripts are recoded. Additionally, a suite of dedicated factors orchestrates this unique translation system. The Sec incorporation process in eukaryotes unfolds through a four-step regulatory cascade: i) UGA recoding: Ribosomes are reprogrammed to recognize UGA as a Sec codon rather than a stop signal. ii) Sec-tRNA^Sec^ recruitment: The specialized tRNA, Sec-tRNA^Sec^, is delivered to the ribosome by selenocysteine-tRNA specific eukaryotic elongation factor (eEFSec).^87^, ^88^, ^89^ iii) SECIS-mediated regulation: The SECIS-binding protein 2 (SBP2) recruits the SECIS element to the ribosome, facilitating UGA reassignment.^87^^,^^90^, ^91^, ^92^, ^93^ iv) Cis-acting elements: The Sec redefinition element (SRE), located upstream of the UGA codon, collaborates with the SECIS to establish a translational context that favors Sec insertion over termination^94^^,^^95^ (Fig. 3). This intricate mechanism ensures the fidelity of Sec incorporation while competing with translational termination, highlighting the sophistication of selenoproteins biosynthesis.^96^ The molecular interplay between SECIS structures, trans-acting factors, and ribosomal components represents a unique solution to the challenge of genetic code expansion in eukaryotic cells.

## Human selenoproteome

Although biochemical approaches have elucidated the biosynthesis of Sec and its incorporation into selenoproteins, the discovery of novel selenoproteins remains a significant challenge in selenoprotein research.^97^ Selenoprotein mRNAs are characterized by two key features: an in-frame UGA codon encoding Sec,^87^ and a *cis*-acting Sec insertion sequence (SECIS) element in the 3′ untranslated region (UTR).^98^ Notably, the presence of a UGA codon alone is insufficient to confirm a selenoprotein, necessitating bioinformatic identification of SECIS elements. The current strategy for selenoproteins discovery involves i) high-throughput sequencing to obtain genomic data, ii) computational analysis using SECIS-prediction software,^99^, ^100^, ^101^ and iii) experimental validation via ^75^Se metabolic labeling of cells.^102^, ^103^, ^104^, ^105^ This approach has led to the identification of all 25 known human selenoproteins.^106^, ^107^, ^108^, ^109^, ^110^

Mammals possess eight GPX isoforms, five of which (GPX1–4 and GPX6) are selenoproteins.^111^ GPX1, the first mammalian selenoprotein identified in 1973, is the most abundant and is highly expressed in the liver and kidneys.^112^, ^113^, ^114^ As an antioxidant enzyme, it utilizes GSH to catalyze the reduction of hydrogen peroxide.^115^, ^116^, ^117^, ^118^ In 1993, GPX2 was cloned from human intestinal epithelial cells and initially termed “gastrointestinal-specific GPX” (GI-GPX) due to its high expression in the digestive tract.^119^, ^120^, ^121^ Subsequent studies revealed its dual role in colorectal cancer: it may suppress oxidative damage-induced carcinogenesis in early stages but promote cancer cell survival in advanced disease.^122^, ^123^, ^124^, ^125^ GPX3, isolated from human plasma in 1987, is the only secreted GPX and is primarily found in extracellular fluids (*e.g.*, plasma, milk).^126^^,^^127^ It mediates a systemic antioxidant role and is encoded by a gene on chromosome 5q23.^128^, ^129^, ^130^, ^131^, ^132^ GPX4, purified from porcine liver in 1982,^133^ is critical for preventing lipid peroxidation and has recently been implicated in regulating ferroptosis.^134^, ^135^, ^136^, ^137^, ^138^ GPX6, identified through bioinformatics, is unique in that it contains Sec in humans but cysteine in rodents, with high expression in olfactory neurons, likely protecting them from oxidative damage.^139^^,^^140^ Mammals express three deiodinases (DIO1–3), discovered between 1991 and 1997,^141^, ^142^, ^143^ which regulate thyroid hormone activation and inactivation via reductive deiodination.^144^, ^145^, ^146^, ^147^ The mammalian thioredoxin reductases (TXNRDs, also known as TrxRs) were first purified in 1999.^148^ All three isoforms (TXNRD1–3) are selenoproteins, rendering the entire TXN system selenium-dependent.^149^ TXNRD1 maintains cytoplasmic/nuclear TXN1 in a reduced state,^150^ while TXNRD2 acts in mitochondria.^151^ TXNRD3, with an N-terminal glutaredoxin domain, is implicated in sperm maturation.^152^, ^153^, ^154^ The functions of other selenoproteins are described in the Table 1.

**Table 1:** The underexplored landscape of selenoproteins.

Protein name	Functional description of proteins
MSRB1	MSRB1: Catalyzes stereospecific reduction of methionine-R-sulfoxide, repairing oxidative damage to methionine residues.^155^, ^156^, ^157^, ^158^
SEP15	SEP15: A conserved eukaryotic selenoprotein with an endoplasmic reticulum (ER)-targeting signal and a TXN-like domain.^159^, ^160^, ^161^
SEPHS2 (SPS2)	SEPHS2 (SPS2): Essential for selenophosphate synthesis, the selenium donor for Sec biosynthesis.^162^^,^^163^
Selenoprotein P (SELENOP)	Selenoprotein P (SELENOP): The only selenoprotein with multiple Sec residues, functioning in selenium transport.^164^^,^^165^
Selenoprotein W (SELENOW)	Selenoprotein W (SELENOW)^166^, ^167^, ^168^ and V (SELENOV)^169^^,^^170^: SELENOV, a testes-specific paralog of SELENOW, contains an N-terminal extension of unknown function.^109^
Selenoprotein T (SELENOT)	Selenoprotein T (SELENOT)^159^^,^^171^^,^^172^: Reported to suppress lipopolysaccharide-induced endothelial apoptosis.^173^
Selenoprotein M (SELENOM)	Selenoprotein M (SELENOM)^174^: An ER-resident protein linked to hepatocellular carcinoma progression.^175^
Selenoprotein H (SELENOH)	Selenoprotein H (SELENOH)^176^: Localized to the nucleus; its knockout induces oxidative stress.
Selenoprotein O (SELENOO)	Selenoprotein O (SELENOO): The largest selenoprotein, localized to mitochondria, with unclear function.^177^, ^178^, ^179^
Selenoprotein I (SELENOI)	Selenoprotein I (SELENOI)^180^: Vertebrate-specific and membrane-associated, though its role remains unknown.^181^
Selenoprotein K (SELENOK) and Selenoproteins S (SELENOS)	Selenoproteins K (SELENOK) and S (SELENOS): Lack defined secondary structures but are implicated in ER-associated degradation (ERAD) and immune responses.^182^, ^183^, ^184^ SELENOK also regulates protein palmitoylation and cardiomyocyte antioxidant defense,^185^ and SELENOK, together with DHHC6, palmitoylates the inositol 1,4,5-triphosphate receptor, maintaining its stable expression and function.^182^^,^^186^
Selenoprotein N (SELENON)	Selenoprotein N (SELENON): An ER protein with strong evolutionary conservation, suggesting a role in muscle differentiation and maintenance^187^, ^188^, ^189^

**Figure 4:**
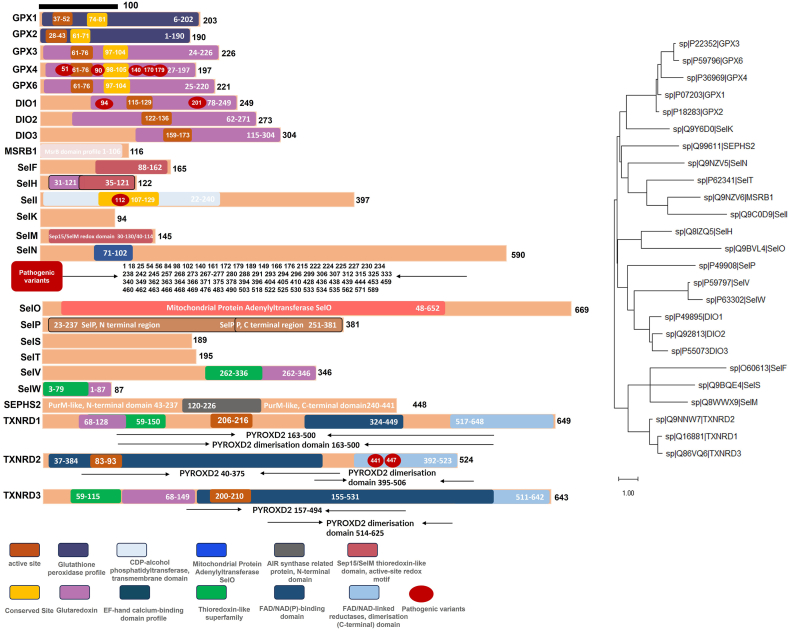
Structural characteristics of 25 human selenoproteins. In the figure, we summarized the structural characteristics and sequence specificity of 25 human selenoproteins. A paraphylogenetic tree was reconstructed with the Jones-Taylor-Thornton (JTT) model-based maximum likelihood method implemented in MEGA11.013 software using the full aligned amino acid sequences of 25 proteins.

This comprehensive overview highlights the structural and functional diversity of mammalian selenoproteins, underscoring their critical roles in redox homeostasis, thyroid metabolism, and cellular signaling (Fig. 4).

## SECIS

**Figure 5:**
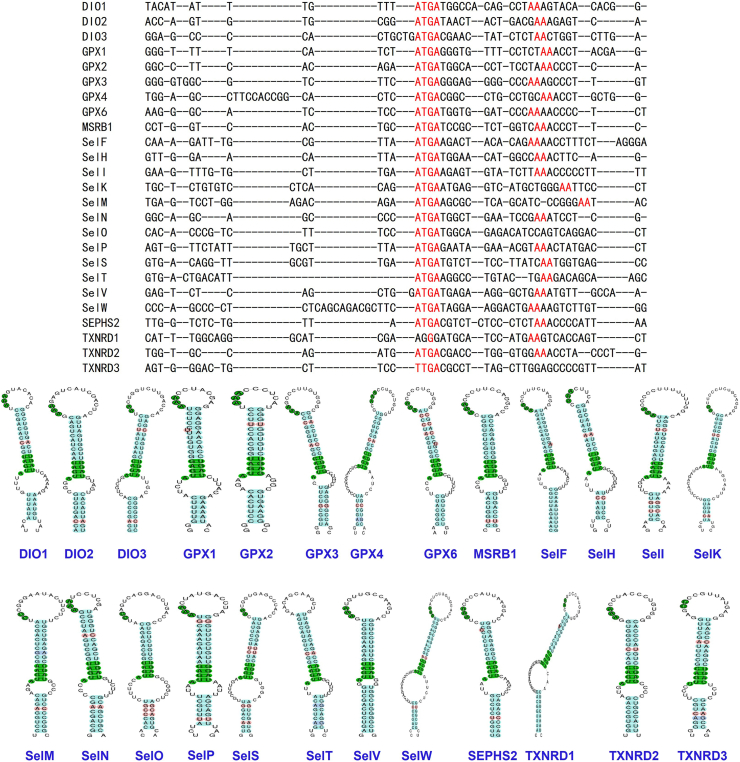
The primary and secondary structures of SECIS for 25 human selenoproteins. In the figure, we compared the SECIS sequences of 25 human selenoproteins and analyzed their secondary structures using the selenoprotein prediction server (https://seblastian.crg.es/).

Incorporation of Sec into selenoprotein translation requires a special mechanism to ensure exclusive recoding of UGA codons in selenoprotein mRNAs, mediated by the SECIS cis-element in the 3′UTR.^190^, ^191^, ^192^ The discovery of the SECIS element originated from studies on DIO1 mRNA.^61^ Initially, researchers observed that the successful Sec incorporation into DIO1 required a specific ∼200-nucleotide sequence in its 3′UTR, which was conserved in both human and rat DIO1 mRNAs. However, this sequence was dispensable for the expression of a cysteine-mutated variant of deiodinase. Furthermore, although the primary sequences of the 3′UTRs of DIO1 and GPX mRNAs shared low similarity, the rat GPX 3′UTR could functionally replace that of DIO1 to direct Sec insertion. Computational analysis revealed that the 3′UTRs of DIO1 and GPX mRNAs could form similar stem-loop secondary structures. These sequences, capable of forming stem-loop structures and guiding Sec incorporation, were termed SECIS.^61^ The SECIS element exhibits highly variable sequences across species and different mRNA 3′UTRs but shares a conserved secondary structure comprising two helices, an internal stem-loop, and an apical loop. SECIS elements are classified into two types based on helical and loop features: Type I features a relatively large apical loop and an internal loop separated by a helix of 12–14 base pairs; Type II contains a short helix (2–7 base pairs), an internal bulge, and a smaller apical loop.^193^ The apical loop harbors conserved AAR residues, while the internal loop contains a conserved SECIS core, which includes an unpaired AUGA and UGR sequence along the 5′ and 3′ ends.^190^ A hallmark structural and functional feature is the AUGA sequence forming a non-Watson-Crick base pair with the 3′ flanking region, creating a kink-turn (K-turn) motif that introduces a ∼120° bend in the helix.^194^^,^^195^ In this study, we summarize the primary and secondary structures of SECIS sequences from all 25 human selenoproteins (Fig. 5).

## The principle of enzymatic activity catalysis of selenoproteins

**Figure 6:**
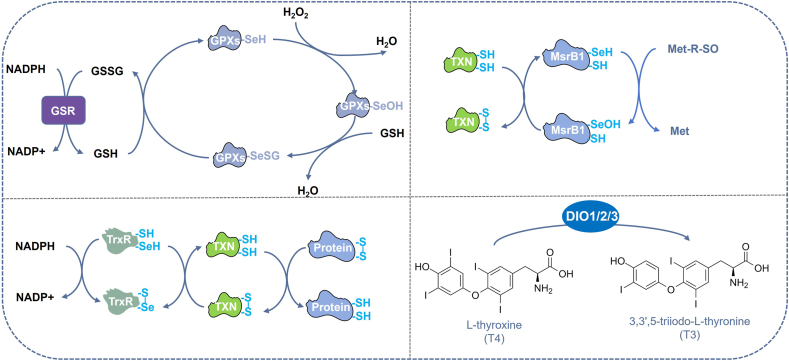
The principle of enzymatic activity catalysis of selenoproteins. In the figure, we summarized their catalytic mechanisms: GPx, TrxR, MSRB1, and DIO. GPx: glutathione peroxidase; TrxR, thioredoxin reductase; MSRB1, methionine-R-sulfoxide reductase B1; DIO, iodothyronine deiodinase.

Selenoproteins, a phylogenetically conserved family of proteins, are distinguished by the incorporation of Sec, a catalytically active amino acid, within their functional domains. This unique residue endows selenoproteins with exceptional enzymatic efficiency, enabling them to execute diverse physiological functions, including antioxidant defense, redox regulation, and metabolic modulation.^196^, ^197^, ^198^ The selenium atom in Sec exhibits stronger nucleophilicity and a lower pKa compared with the sulfur atom in cysteine, conferring superior catalytic efficiency.^199^ Among the 25 human selenoproteins, only the glutathione peroxidase (GPx) family, thioredoxin reductase (TrxR) family, iodothyronine deiodinase (DIO) family, and methionine sulfoxide reductase B1 (MSRB1) display classical enzymatic characteristics. The selenol group (-SeH) of the Sec residue in GPx directly reduces peroxides, forming a selenenic acid intermediate, which is subsequently regenerated by GSH to complete the catalytic cycle.^200^^,^^201^ In TrxR, the C-terminal Sec residue mediates the reduction of oxidized thioredoxin (TXN). The catalytic mechanism involves NADPH transferring electrons to the FAD domain of TrxR, reducing its active-site selenosulfide bond. The reduced TrxR then transfers electrons to the CXXC motif of TXN, converting it from the oxidized (TXN-S_2_) to the reduced (TXN-(SH)_2_) state. Finally, reduced TXN delivers electrons to target proteins, reducing their disulfide bonds.^202^, ^203^, ^204^, ^205^, ^206^, ^207^ As a selenoprotein, MSRB1 utilizes its Sec residue to efficiently repair oxidized methionine residues, protecting proteins from oxidative damage.^208^, ^209^, ^210^, ^211^, ^212^, ^213^ The Sec residue in DIO enzymes participates in thyroid hormone activation through its unique redox properties.^145^^,^^214^, ^215^, ^216^, ^217^, ^218^ This review highlights the catalytic principles of these key selenoproteins, emphasizing the critical role of Sec in their enzymatic functions (Fig. 6).

## Selenoproteins and ferroptosis

Selenoproteins, as a unique family of proteins containing Sec, play pivotal roles in cellular antioxidant defense systems, particularly in the regulation of ferroptosis. This review systematically elucidates the molecular mechanisms by which selenoproteins such as GPX4,^134^^,^^136^^,^^138^^,^^219^, ^220^, ^221^, ^222^, ^223^, ^224^ TrxR,^225^, ^226^, ^227^, ^228^, ^229^, ^230^, ^231^, ^232^, ^233^, ^234^, ^235^, ^236^ and SELENOI^237^ defend against ferroptosis. We comprehensively discuss the intricate regulatory networks governing selenoproteins at transcriptional, translational, post-translational modification, and enzymatic activity levels, while categorizing ferroptosis modulators targeting selenoproteins. These findings provide crucial theoretical foundations for understanding the molecular basis of ferroptosis and developing therapeutic strategies for related diseases.

As the central selenoproteins in ferroptosis defense,^219^^,^^221^^,^^238^, ^239^, ^240^, ^241^, ^242^, ^243^ GPX4 exists in three isoforms generated through alternative splicing from a single gene.^244^ i) Cytosolic GPX4 (cGPX4, 20 kDa)^245^: Essential for embryonic development, as its knockout results in embryonic lethality.^246^, ^247^, ^248^ cGPX4 can translocate across the outer mitochondrial membrane and accumulate in the intermembrane space to suppress lipid peroxidation.^249^ Studies demonstrate that cGPX4 overexpression effectively rescues embryonic fibroblast death induced by GPX4 knockout.^250^^,^^251^ ii) Mitochondrial GPX4 (mGPX4, 23 kDa): Contains an N-terminal mitochondrial targeting signal and is predominantly expressed in sperm cells.^252^ mGPX4 deficiency causes male infertility in mice,^253^ primarily by inhibiting mitochondrial cardiolipin peroxidation.^250^^,^^254^, ^255^, ^256^ As a major component of the inner mitochondrial membrane rich in polyunsaturated fatty acids, cardiolipin is highly susceptible to oxidation.^257^, ^258^, ^259^ Oxidized cardiolipin reduces binding affinity for cytochrome C, triggering cytochrome C release and apoptosis initiation. mGPX4 maintains cardiolipin in a reduced state to prevent this process.^244^^,^^260^ iii) Nuclear GPX4 (nGPX4, 34 kDa)^261^: Primarily expressed in late spermatids and spermatozoa, playing a critical role in stabilizing sperm chromatin structure.^250^^,^^262^^,^^263^

GPX4 utilizes GSH as a reducing equivalent to convert lipid peroxides into non-toxic lipid alcohols, with its catalytic activity dependent on the Sec residue at the active site.^264^, ^265^, ^266^, ^267^ This antioxidant system comprises three key components: i) Cystine uptake and metabolism: The xCT system, composed of solute carrier family 7 member 11 (SLC7A11) and solute carrier family 3 member 2 (SLC3A2), mediates cystine/glutamate antiport at a 1:1 ratio.^268^, ^269^, ^270^, ^271^, ^272^, ^273^, ^274^ Imported cystine is reduced to cysteine by TrxR1, an important selenoprotein that maintains cellular redox homeostasis via the TXN system and plays an auxiliary role in ferroptosis defense.^233^^,^^275^^,^^276^ ii) GSH synthesis: Cysteine serves as the rate-limiting substrate for GSH synthesis, catalyzed by glutamate-cysteine ligase (GCL)^277^, ^278^, ^279^, ^280^ and glutathione synthetase (GSS)^281^, ^282^, ^283^ in conjunction with glutamate and glycine. iii) Antioxidant function of GPX4: GPX4 employs GSH to reduce lipid peroxides, thereby terminating the lipid peroxidation chain reaction.^224^^,^^242^^,^^267^^,^^284^, ^285^, ^286^, ^287^, ^288^, ^289^, ^290^, ^291^

**Figure 7:**
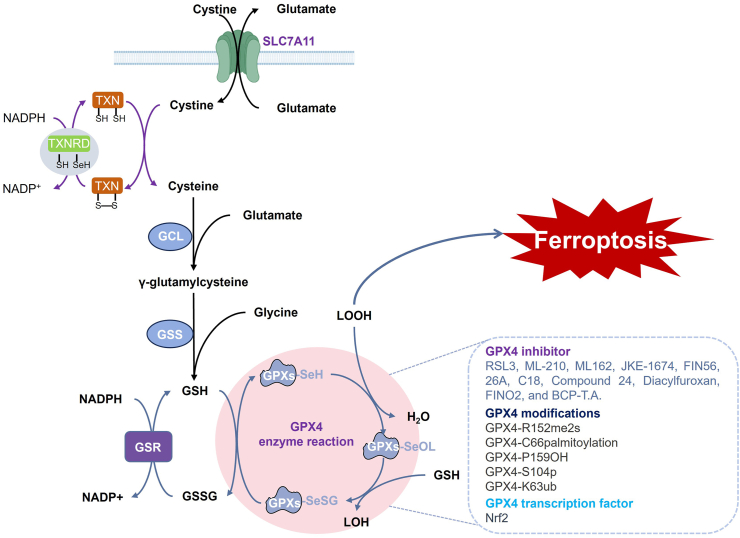
The mechanism of GPX4 in combating ferroptosis. In the figure, we described the mechanism by which GPX4 defends against ferroptosis, as well as its inhibitors, post-translational modifications, and transcriptional regulators.

As a key selenoprotein defending against ferroptosis, GPX4 is extensively regulated at multiple levels. i) Transcriptional regulation: In hepatocellular carcinoma, glutathione S-transferase zeta 1 (GSTZ1) deficiency leads to succinylacetone accumulation, which alkylates kelch-like ECH-associated protein 1 (KEAP1) and activates nuclear factor erythroid 2-related factor 2 (Nrf2), subsequently up-regulating GPX4 expression.^292^^,^^293^ Conversely, protein arginine methyltransferase 4 (PRMT4) methylates Nrf2 to inhibit its nuclear translocation, thereby suppressing GPX4 expression.^294^^,^^295^ Inhibition of kinesin family member 2A (KIF2A) suppresses NUAK family kinase 1 (NUAK1) activation, up-regulates protein phosphatase 1 beta (PP1β) expression, reduces glycogen synthase kinase 3 beta (GSK3β) Ser9 phosphorylation, and suppresses the nuclear import and transcription activity of Nrf2, ultimately inhibiting the expression of GPX4, sensitizing cells to drug-induced ferroptosis.^296^ The gut microbial metabolite capsiate up-regulates GPX4 expression through transient receptor potential cation channel subfamily V member 1 (TRPV1) activation.^297^ Kruppel-like factor 2 (KLF2), a zinc finger-containing transcription factor, binds to the −1057/–1046 promoter region of GPX4 to repress its transcription.^298^ Homocysteine (Hcy) induces expression of DNA methyltransferases (DNMT1, DNMT3a, and DNMT3b), increasing GPX4 gene methylation in nucleus pulposus cells and consequently reducing GPX4 mRNA and protein levels.^299^, ^300^, ^301^ ii) Translational regulation: As a selenoprotein, GPX4 translation is precisely regulated by selenium availability. Selenium deficiency significantly suppresses GPX4 expression and promotes ferroptosis, while selenium supplementation up-regulates GPX4 levels to inhibit ferroptosis.^302^, ^303^, ^304^ In addition, the xCT-mediated cystine uptake not only facilitates GSH synthesis but also directly regulates GPX4 expression through the recombination activating gene (Rag)–mechanistic target of rapamycin complex 1 (mTORC1)–4E-binding protein (4EBP) signaling axis.^137^^,^^305^^,^^306^ iii) Post-translational modifications and degradation: Legumain promotes heat shock cognate protein 70 (HSC70)/heat shock protein 90 (HSP90)-mediated autophagic degradation of GPX4.^307^^,^^308^ Erastin increases lysosomal-associated membrane protein 2A (LAMP2A) expression to enhance chaperone-mediated autophagy and GPX4 degradation.^309^ FUN14 domain-containing 1 (FUNDC1) recruits GPX4 to mitochondria through the translocase of the outer membrane (TOM)/translocase of the inner membrane (TIM) complexes for PTEN-induced kinase 1 (Pink1)/Parkin-mediated mitophagic degradation.^310^ The E3 ubiquitin ligase ring finger and CCCH-type domains 1 (RC3H1) ubiquitinates GPX4 to promote its degradation, while mucosa-associated lymphoid tissue lymphoma translocation 1 (MALT1) cleaves RC3H1 to stabilize GPX4.^311^ Protein arginine methyltransferase 5 (PRMT5) catalyzes GPX4 methylation to block T40/S44 phosphorylation, preventing F-box and WD40 repeat domain containing-7 (FBW7)-mediated ubiquitination.^312^^,^^313^ Phosphoserine aminotransferase 1 (PSAT1) generates α-ketoglutarate (α-KG) to promote prolyl hydroxylase domain protein 3 (PHD3)-mediated GPX4 hydroxylation, inhibiting HSC70 binding and stabilizing GPX4.^314^ ZDHHC palmitoyltransferase 20 (ZDHHC20) enhances GPX4 stability through palmitoylation, whereas Acyl-protein thioesterase 2 (APT2)-mediated depalmitoylation has the opposite effect.^315^^,^^316^ Under conditions of insulin-like growth factor 1 receptor (IGF1R) activation, creatine kinase B (CKB) is phosphorylated at threonine 133 (T133) by protein kinase B (PKB or AKT), leading to the suppression of its canonical metabolic enzyme activity. Subsequently, CKB acquires a non-canonical protein kinase function, enabling it to bind to and phosphorylate GPX4 at serine 104 (S104), and this phosphorylation prevents HSC70 binding to GPX4, thereby abrogating the GPX4 degradation regulated by chaperone-mediated autophagy, alleviating ferroptosis, and promoting tumor growth in mice.^317^ Given GPX4's crucial role in ferroptosis inhibition, developing GPX4-targeted inhibitors has become a major focus in ferroptosis research.^318^ Currently identified GPX4 inhibitors include RSL3,^136^^,^^319^, ^320^, ^321^, ^322^, ^323^ ML-210,^319^^,^^324^^,^^325^ ML162,^319^^,^^326^^,^^327^ JKE-1674,^328^, ^329^, ^330^ FIN56,^331^, ^332^, ^333^ A16,^334^ 26a,^335^ C18,^336^ compound 24,^337^ diacylfuroxan,^338^ FINO_2_,^224^ and BCP-T.A^339^ (Fig. 7).

In conclusion, selenoproteins GPX4 and TrxR occupy central positions in the ferroptosis defense system, maintaining cellular redox balance through sophisticated multi-level regulatory networks. From transcriptional control to post-translational modifications, cells have evolved precise mechanisms to regulate selenoproteins expression and activity. Small-molecule modulators targeting selenoproteins, particularly natural compounds, demonstrate therapeutic potential across various disease models.

## Selenoproteins and apoptosis

Apoptosis, a form of programmed cell death,^340^^,^^341^ is a ubiquitous biological process tightly regulated by intracellular signaling molecules.^340^ Characterized by distinct morphological changes, it involves a cascade of complex biochemical reactions culminating in cell fragmentation into apoptotic bodies,^342^ which are rapidly phagocytosed by macrophages or neighboring cells without eliciting an inflammatory response.^343^, ^344^, ^345^ Recent studies have revealed a close relationship between selenoproteins and apoptosis, demonstrating that selenoproteins can inhibit apoptosis contingent upon cell type, specific selenoproteins involved, and cellular microenvironment. Certain selenoproteins, such as the GPX1–4^346^, ^347^, ^348^, ^349^, ^350^, ^351^, ^352^, ^353^ and TrxR1–3,^354^, ^355^, ^356^, ^357^ play a pivotal role in suppressing apoptosis by maintaining redox homeostasis through scavenging reactive oxygen species (ROS). Excessive ROS accumulation triggers mitochondrial membrane potential collapse, leading to cytochrome C release and subsequent activation of the caspase cascade, ultimately inducing apoptosis.^358^ GPX and TrxR enzymes mitigate this process by degrading hydrogen peroxide (H_2_O_2_) and lipid peroxides, thereby blocking ROS-mediated apoptotic signaling.

SELENOK gene knockout markedly enhanced endoplasmic reticulum stress,^359^ promoting apoptosis in neurons via intracellular Ca^2+^ flux and activation of the m-calpain/caspase-12 cascade, both *in vivo* and *in vitro*.^360^ Similarly, SELENOK knockdown induces apoptosis in skeletal muscle satellite cells through calcium dyshomeostasis-mediated endoplasmic reticulum stress.^361^^,^^362^ SELENOT protects against cisplatin-induced acute kidney injury by suppressing oxidative stress and apoptosis.^172^ Research shows that iodothyronine deiodinase 2 (DIO2) is expressed in cytotrophoblasts, proximal column trophoblasts, distal column trophoblasts, and syncytiotrophoblasts of the placenta. Overexpression of DIO2 arrested trophoblast cell proliferation at the G1 phase of the cell cycle by down-regulating cyclin D1 and proliferating cell nuclear antigen (PCNA), while simultaneously promoting apoptosis through enhanced caspase-3 activity and inhibition of the AKT and extracellular signal-regulated kinase 1/2 (ERK1/2) signaling pathways, and its down-regulation is associated with early recurrent miscarriage.^363^ MsrB1 gene silencing by short interfering RNA (siRNA) independently resulted in oxidative stress, endoplasmic reticulum stress, activation of caspase-3, and an increase of apoptosis in HLE cells,^364^ and MsrB1 gene silencing by siRNA in HLE cells clearly resulted in oxidative stress, decrease in mitochondrial membrane potential, and release of mitochondrial cytochrome C, as well as an increase in activity of caspase-3 and the percentage of apoptotic cells.^365^

In summary, selenoproteins influence apoptosis through redox regulation, endoplasmic reticulum stress modulation, and signaling pathway interference, with their effects being highly context-dependent. Elucidating these mechanisms may provide novel therapeutic targets for various diseases.

## Selenoproteins and other cell death

Cuproptosis, a newly identified form of regulated cell death, is triggered by intracellular copper accumulation. Specifically, excessive copper induces aggregation of mitochondrial lipoylated proteins and destabilization of iron-sulfur cluster proteins, ultimately leading to cell death.^366^^,^^367^ Studies have reported that GSH exhaustion via inhibition of the xCT–GSH–GPX4 pathway synergistically enhances DSF/Cu-induced cuproptosis in myelodysplastic syndromes.^368^ Furthermore, exogenous copper promotes GPX4 ubiquitination and aggregate formation by directly binding to cysteine residues C107 and C148 on GPX4. Subsequently, Tax1-binding protein 1 (TAX1BP1) serves as an autophagic receptor for GPX4 degradation, driving ferroptosis under copper stress.^369^

Disulfidptosis is a novel cell death mechanism mediated by SLC7A11, induced by disulfide stress (*e.g.*, sulfur dioxide, hydrogen sulfide) from excessive intracellular cystine accumulation.^370^^,^^371^ Research has demonstrated that synchronously inducing disulfidptosis and ferroptosis through systematic glucose deprivation targets the SLC7A11/GSH/GPX4 antioxidant axis.^372^

Pyroptosis, a form of programmed necrotic cell death, is triggered by intracellular infections involving bacteria, viruses, fungi, or protozoa, in response to pathogen-associated molecular patterns (PAMPs) or damage-associated molecular patterns (DAMPs).^373^, ^374^, ^375^ This process is primarily induced in innate immune cells, such as monocytes, macrophages, and dendritic cells, and is characterized by inflammasome activation, gasdermin-mediated pore formation in the plasma membrane, and the release of pro-inflammatory cytokines.^376^, ^377^, ^378^, ^379^ Emerging evidence highlights selenoproteins as regulators of pyroptosis. GPX4, a key antioxidant enzyme, suppresses pyroptosis by reducing lipid peroxidation, thereby protecting against septic lethality in mice.^380^^,^^381^ Similarly, GPX3 inhibits microglial pyroptosis via the interleukin-1 receptor-associated kinase 4 (IRAK4)/ROS/NLR family pyrin domain containing 3 (NLRP3) pathway, mitigating spinal cord injury.^382^ Conversely, selenoprotein W promotes hepatocyte apoptosis and pyroptosis by regulating metabolic reprogramming to activate cyclic GMP-AMP synthase (cGAS)/stimulator of interferon genes (STING) signaling in macrophages, thereby exacerbating the progression of nonalcoholic fatty liver disease.^168^ Selenoprotein O ablation enhances neutrophil recruitment and enhances ROS-dependent neutrophil extracellular trap formation by increasing high-mobility group box 1 (HMGB1) expression, thereby aggravating lipopolysaccharide-induced pyroptosis and inflammation.^383^ Additionally, selenium supplementation up-regulates GPX4, counteracting cadmium- or arsenic-induced pyroptosis and ferroptosis.^384^ These findings underscore the dual role of selenoproteins in pyroptosis regulation, offering potential therapeutic targets for inflammatory and metabolic disorders.

Necroptosis, a regulated form of cell death, is initiated when apoptosis is inhibited, triggered by extracellular signals (*e.g.*, death receptor–ligand interactions) or intracellular stimuli (*e.g.*, microbial nucleic acids).^385^, ^386^, ^387^, ^388^ Accumulating evidence shows that selenium deficiency promotes necroptosis,^389^, ^390^, ^391^, ^392^, ^393^, ^394^, ^395^ whereas certain selenoproteins, including GPX4,^247^ TrxR,^396^ selenoprotein S,^397^ and selenoprotein K,^398^ exert protective effects. However, the roles of other selenoproteins in necroptosis regulation remain undefined, warranting further investigation. Elucidating these mechanisms may provide novel therapeutic strategies for diseases involving dysregulated necroptotic pathways.

## Selenoproteins and cancer

**Figure 8:**
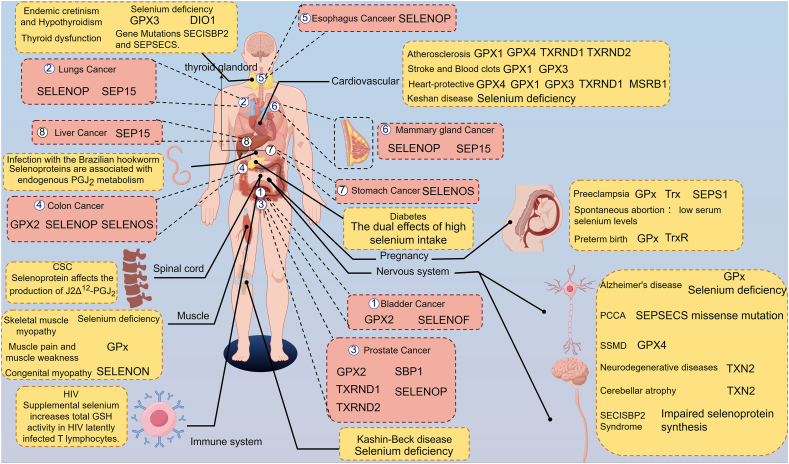
Selenoproteins and related diseases. In the figure, we summarized the role of selenoproteins in cancer and other diseases. This figure was created with figdraw 2.0 (https://www.figdraw.com/).

Rapidly proliferating tumor cells face three major challenges: i) high energy demands to sustain proliferation, ii) increased biosynthesis of macromolecules,^399^ and iii) elevated oxidative stress.^400^, ^401^, ^402^, ^403^, ^404^, ^405^, ^406^, ^407^ To counteract oxidative damage, malignant tumors rely on a robust antioxidant system.^408^ Selenoproteins, which protect both tumors and normal cells from oxidative stress, exhibit a dual role in cancer progression, functioning as suppressors or promoters depending on tissue and cellular context^409^ (Fig. 8).

## GPX1/2/3/4/6

As a key intracellular antioxidant enzyme, GPX1 maintains redox homeostasis by scavenging reactive ROS. Its role in cancer is highly tissue-specific. i) Tumor-suppressive effects: In pancreatic cancer, down-regulation of GPX1 is associated with poor prognosis.^410^ Furthermore, genetic or functional ablation of GPX1 promotes epithelial–mesenchymal transition and confers chemoresistance through activation of ROS-mediated Akt/GSK3β/Snail signaling.^411^ ii) Pro-tumorigenic effects: In triple-negative breast cancer, GPX1 promotes tumor migration and metastasis via activation of the focal adhesion kinase (FAK) signaling^412^; in non-small cell lung cancer, it enhances cisplatin resistance through ROS-induced activation of phosphoinositide 3-kinase (PI3K)/AKT pathway^413^; in receptor-interacting serine/threonine kinase 3 (RIPK3)-negative cancer cells, GPX1 regulates apoptosis signal-regulated kinase 1 (ASK1)-dependent apoptosis via interaction with tumor necrosis factor receptor-associated factor 2 (TRAF2).^348^ GPX2 is highly expressed in gastrointestinal, breast, bladder, and lung epithelial cells, with stage-dependent functions. i) Early protective role: By scavenging hydrogen peroxide, GPX2 reduces DNA damage and may prevent inflammation-associated carcinogenesis.^122^ ii) Pro-tumorigenic mechanisms: In established tumors, GPX2 supports tumor progression by acting as a Wnt pathway target gene to regulate cancer stem cell proliferation.^414^, ^415^, ^416^, ^417^ GPX2 knockdown suppresses gastric cancer progression and metastasis via regulation of kynurenine metabolism,^418^ while GPX2 loss drives malignant progression through ROS/hypoxia inducible factor-α (HIF1α)/vascular endothelial growth factor A (VEGFA) signaling, causing poor perfusion and hypoxia reversed by GPX2 re-expression or HIF1α inhibition.^419^ Notably, GPX2 is a metabolic driver of the tumor immune microenvironment and immune checkpoint inhibitor response.^420^ GPX3 is the only secretory GPX that exhibits a paradoxical “plasma-tissue” expression pattern in cancer. i) Plasma levels: In hepatocellular carcinoma, low plasma GPX3 correlates with advanced tumor stage and worse prognosis.^128^^,^^421^, ^422^, ^423^ ii) Tissue expression: In serous ovarian cancer, high tumor GPX3 expression associates with malignant progression and poor outcomes, implicating a role in tumor microenvironment remodeling.^424^^,^^425^ As a ferroptosis inhibitor, GPX4 overexpression is linked to therapy resistance in multiple cancers.^224^^,^^426^, ^427^, ^428^, ^429^, ^430^, ^431^, ^432^, ^433^, ^434^, ^435^ Pharmacological inhibition of GPX4 selectively induces ferroptosis in tumor cells, emerging as a promising therapeutic strategy.^135^^,^^226^^,^^333^^,^^369^^,^^436^, ^437^, ^438^, ^439^, ^440^, ^441^, ^442^, ^443^, ^444^, ^445^, ^446^, ^447^, ^448^, ^449^, ^450^, ^451^, ^452^ Primarily expressed in the olfactory epithelium and testis, GPX6 is up-regulated in gastric cancers,^453^ and its down-regulation promotes tumor progression via oxidative stress accumulation.^454^^,^^455^

## TrxR1/2

The TrxR system plays a critical role in cancer development, and TrxR inhibition is generally considered beneficial for suppressing tumor growth.^206^^,^^207^^,^^456^, ^457^, ^458^, ^459^, ^460^, ^461^ The C-terminal Sec residue of TrxR1 is highly reactive and represents a valuable target for drug development. Many clinically used anti-cancer agents inhibit TrxR1, impairing endogenous antioxidant defenses and elevating ROS to induce cancer cell death.^461^, ^462^, ^463^, ^464^, ^465^, ^466^, ^467^, ^468^, ^469^, ^470^, ^471^ Mitochondrial TrxR2 is essential for tumor growth and angiogenesis.^472^^,^^473^ Inhibition of TrxR2 suppressed non-small cell lung cancer cell proliferation and metabolism and induced apoptosis via decreasing antioxidant activity.^474^

## SEPHS2

An essential enzyme in the selenocysteine biosynthesis pathway, SEPHS2 is crucial for cancer cell survival.^163^^,^^475^ Cancer cells critically depend on SEPHS2 to detoxify selenide, an intermediate in selenocysteine biosynthesis.^476^ Through the secondary function of the cystine/glutamate antiporter SLC7A11, which facilitates selenium uptake and selenocysteine biosynthesis, breast cancer and other cancer cells autonomously enable the production of selenoproteins (such as GPX4) to evade ferroptosis. However, since selenide is inherently toxic, its processing by SEPHS2 becomes an obligate dependency for cancer cells. Clinical studies have demonstrated elevated SEPHS2 protein levels in breast cancer patient samples, and its genetic ablation significantly impairs the growth of orthotopic mammary tumor xenografts in mouse models.

## MSRB1

Implicated in tumorigenesis and immune regulation, MSRB1 is frequently overexpressed in tumor tissues.^157^^,^^477^^,^^478^ MSRB1 appears to modulate the tumor immune microenvironment, potentially influencing cancer progression, and may serve as a novel regulator of anti-tumor immunity.^479^

## Other selenoproteins

Related research is currently focused on colon and prostate cancers,^480^, ^481^, ^482^, ^483^, ^484^, ^485^ and autoimmunity to selenoprotein P (SELENOP) predicts breast cancer recurrence.^486^ Selenoprotein S (SELENOS) is a small, intrinsically disordered membrane protein primarily known for its contribution to governing the extraction of misfolded proteins or misassembled protein complexes from the endoplasmic reticulum to the cytosol for degradation by the proteasome. It is associated with various cellular functions, such as inflammatory processes, cellular stress response, protein quality control, and signaling pathway.^487^ SELENOS knockdown sensitized colorectal cancer cells to ROS-mediated anti-tumor effects of regorafenib,^488^ and serves as a potential prognostic biomarker for brain lower-grade glioma.^489^ Selenoprotein 15 (SEP15) is an endoplasmic reticulum-resident oxidoreductase involved in protein quality control. SEP15 deficiency inhibits human colon cancer cell growth.^490^ Lung cancer risk associated with selenium status is modified in smoking individuals by the SEP15 polymorphism.^491^ SEP15 (also called SELENOF)^492^ expression is altered in lung cancer patients and male bladder cancer patients,^493^ and enhancing SELENOF expression reduces tumor growth in breast cancer xenografts.^494^^,^^495^ Research shows that a dose-dependent depletion of the Ca^2+^ pool under the action of selenoprotein M (SELENOM), which proves the important role of this protein in the regulation of calcium homeostasis in the cell.^496^ Selenoprotein O (SELENOO) deficiency limits melanoma metastasis by modulating mitochondrial function and oxidative stress.^178^ Inhibition of selenoprotein I (SELENOI) promotes ferroptosis and reverses resistance to platinum chemotherapy by impairing Akt phosphorylation in ovarian cancer.^497^ Selenoprotein K (SELENOK) is an endoplasmic reticulum-resident protein that regulates endoplasmic reticulum stress, calcium flux, and antioxidant defense.^186^ SELENOK knockdown shrinks tumor by modulating ferroptosis, offering a theoretical basis for cervical cancer therapy.^498^

Selenoproteins, containing selenium in the form of Sec, exhibit dual roles in tumorigenesis. Antioxidant selenoproteins like GPXs and TrxRs protect against cancer by reducing oxidative stress, while others promote progression through context-dependent mechanisms. Elucidating these functions may unlock novel therapeutic strategies for cancer.

## Limitations and perspectives

The exploration of selenoproteins has unearthed profound insights into their distinctive translational mechanisms and pivotal roles in modulating cell death pathways and cancer therapeutic strategies. Nevertheless, several significant limitations persist. Primarily, the intricate regulatory mechanisms governing Sec incorporation into selenoproteins remain incompletely elucidated, especially the competitive dynamics between UGA recoding for Sec insertion and translational termination. Although SECIS elements and associated trans-acting factors, such as SBP2 and eEFSec, are recognized for their facilitation of Sec insertion, the dynamic regulation of this process under diverse physiological and pathological conditions, including selenium deficiency, oxidative stress, or cancer progression, warrants in-depth investigation. Moreover, the functional redundancy and compensatory mechanisms among selenoproteins, exemplified by GPX isoforms and TrxRs, complicate the interpretation of genetic and pharmacological studies. For example, while GPX4 is a well-established ferroptosis suppressor, the contributions of other selenoproteins, such as TrxR1 and SELENOK, to ferroptosis and other forms of regulated cell death remain inadequately characterized.

Another major challenge resides in the translation of selenoproteins research into clinical applications. Despite the promising preclinical outcomes of targeting selenoproteins, such as the induction of ferroptosis through GPX4 inhibition in cancer models, several obstacles must be addressed.

In the field of cancer prevention, current clinical trial results indicate that the selenium supplement selenomethionine does not exhibit preventive effects against most types of cancer and may even promote carcinogenesis. A clinical study by Eric A Klein et al showed that, compared with the placebo group, the selenomethionine group had an increased prostate cancer risk of 0.8 per 1000 person-years.^499^ Research by Paul J Limburg et al demonstrated that after 10 months of intervention, selenomethionine failed to inhibit esophageal squamous cell carcinoma in all high-risk subjects.^500^ Moreover, a clinical trial by Lance et al suggested that selenium supplementation is not recommended for the prevention of colorectal adenomas.^501^ In addition, research by Alan R Kristal et al indicated that selenium supplements provide no benefit to men with low selenium status but increase the risk of high-grade prostate cancer in those with high selenium levels.^502^ The Selenium and Vitamin E Cancer Prevention Trial (SELECT) trial enrolled healthy North American males whose average baseline selenium levels were already sufficient or even elevated. Under these conditions, additional supplementation in the form of selenomethionine may further increase bodily selenium content from sufficient to excessive levels, thereby negating potential benefits and possibly causing harm.

In the context of cancer treatment, the selenium compound sodium selenite may serve as an adjuvant therapeutic agent to improve clinical outcomes. Clinical research by Inas A Asfour et al indicated that administration of sodium selenite synergizes with chemotherapy to induce lymphoma cell death, while also exerting a cardioprotective effect in non-Hodgkin lymphoma patients undergoing chemotherapy, thereby enhancing treatment efficacy.^503^ Studies by Thomas Zimmermann et al showed that sodium selenite treatment helps reduce postoperative lymphedema in patients undergoing oral cancer surgery.^504^ A clinical trial by Kiremidjian-Schumacher et al demonstrated that oral administration of sodium selenite enhances immune function in head and neck cancer patients receiving treatments including surgery, radiotherapy, or combined modalities.^505^

To date, relatively few clinical trials have been conducted on selenoprotein-targeting regulators. Clinical trials for the selenoprotein GPX4-targeted inhibitors RSL3 and ML162 have not yet been initiated. RSL3 exhibits toxicity toward normal cells and unfavorable pharmacokinetic properties, which limit its clinical applicability.^506^ Although RSL3 can effectively induce ferroptosis in various tumor cells, its potential damage to normal tissues requires further optimization. Delivery of RSL3 via nanocarriers (*e.g.*, PLGA-PEG^507^ or Fe-EGCG@RSL3^508^) may improve targeting efficiency and reduce side effects; however, most of these technologies remain in early-stage development, and no reliable delivery strategy is sufficiently mature to support clinical trials of RSL3. Furthermore, research on ML162 is still inadequate, and its preclinical performance does not yet meet the requirements for clinical translation.

Future research endeavors should center on three key aspects: i) Mechanistic elucidation: Leveraging advanced structural biology techniques, such as cryo-EM, to decipher the Sec incorporation machinery and employing CRISPR-based screening to identify novel regulators of selenoproteins expression; ii) Therapeutic optimization: Developing tissue-specific selenium delivery systems, such as nanoparticle-based carriers and next-generation selenoprotein inhibitors with improved pharmacokinetics; and iii) Translational exploration: Investigating selenoproteins-based biomarkers, such as plasma GPX3 and SELENOP autoantibodies, for early cancer detection and prognosis. By addressing these challenges, selenoproteins research holds the potential to unlock innovative strategies for precision oncology, particularly in cancers refractory to conventional therapies.

## Conclusions

Selenoproteins constitute a fascinating class of proteins, with their unique biosynthesis and functional versatility highlighting their indispensable roles in maintaining cellular redox homeostasis, regulating cell death, and influencing cancer biology. The Sec insertion mechanism, mediated by SECIS elements and specialized translation factors, represents an elegant evolutionary adaptation that endows these proteins with irreplaceable functions, ranging from antioxidant defense (GPXs, TrxRs) to thyroid hormone metabolism (DIOs). Notably, selenoproteins act as double-edged swords in the context of cancer, functioning as tumor suppressors by alleviating oxidative damage or as oncogenic facilitators by promoting cancer cell survival. The discovery of GPX4's central role in ferroptosis has revolutionized our understanding of selenoproteins in cell death regulation, opening up new therapeutic frontiers for inducing ferroptosis in therapy-resistant cancers.

However, the intricate regulatory landscape of selenoproteins, which encompasses transcriptional, translational, and post-translational levels, necessitates further exploration to fully exploit their therapeutic potential. Future studies should prioritize clarifying the context-dependent functions of selenoproteins across various cancer types, optimizing selenoproteins-targeted therapies, and elucidating their interactions with the tumor microenvironment. Ultimately, a more profound understanding of selenoproteins will not only propel fundamental biology research forward but also lay the groundwork for innovative precision medicine treatments, especially for cancers characterized by oxidative stress and dysregulated cell death pathways.

## CRediT authorship contribution statement

**Chaoyi Xia:** Writing – review & editing, Writing – original draft, Funding acquisition. **Yifan Wu:** Writing – original draft. **Haoming Zhang:** Writing – original draft. **Lang Qin:** Writing – original draft. **Yiren Hu:** Writing – review & editing. **Caiyun Fu:** Writing – review & editing, Funding acquisition.

## Funding

This work was supported by the 10.13039/501100004731Zhejiang Provincial Natural Science Foundation of China (No. LD22H310004, LQN25C070001) and Zhejiang Sci-Tech University Research Start-up Fund (China) (No. 24042191-Y).

## Conflict of interests

Caiyun Fu is the member of *Genes & Diseases* Editorial Board. To minimize bias, she was excluded from all editorial decision-making related to the acceptance of this article for publication. The remaining authors declare no conflict of interests.
